# Magnetic Zinc Oxide/Manganese Ferrite Composite for Photodegradation of the Antibiotic Rifampicin

**DOI:** 10.3390/ma15228185

**Published:** 2022-11-18

**Authors:** Filipe da Silva Duarte, Amanda Lys Matos dos Santos Melo, Alice de Barros Ferro, Carmem Lúcia de Paiva e Silva Zanta, José Leandro da Silva Duarte, Rosane Maria Pessoa Betânio Oliveira

**Affiliations:** 1 Postgraduate Program in Materials Science and Engineering, Federal University of Sergipe, Sergipe 49100-000, Brazil; 2 Chemistry and Biotechnology Institute, Federal University of Alagoas, Maceió 57072-970, Brazil

**Keywords:** photocatalysis, rifampicin, zinc oxide, manganese ferrite, ZnO/MnFe_2_O_4_

## Abstract

In this study, a composite of zinc oxide and manganese ferrite was synthesized using co-precipitation and hydrothermal routes, to be used as photocatalysts in reactions with UV/Vis light source. The synthesized materials were characterized by FTIR, XRD, and SEM, where it was possible to verify the efficiency of the syntheses performed, through the identification of the resulting phases, the evaluation of the structural morphology of the particles, and the analysis of the detachments of the main vibration bonds present in these materials. The composite ZnO/MnFe_2_O_4_ was used in photodegradation reactions of the antibiotic rifampicin, with catalyst dosage of 0.20; 0.40, and 0.60 g and 10 ppm of rifampicin, reactions using pure ZnO as a catalyst were also performed as a comparative parameter of the influence of MnFe_2_O_4_ in this system. The composite ZnO/MnFe_2_O_4_ showed a maximum percentage of rifampicin decontamination of 94.72% and ZnO, 74.20%using 0.20 g of photocatalyst after 90 min, which indicates a positive influence on this process. The solution treated with ZnO/MnFe_2_O_4_ was subjected to magnetic field induction for attraction and consequently accelerated removal of the solids present, successfully, compacting for the application of ZnO/MnFe_2_O_4_ to be presented as a promising material for decontamination of emerging pollutants through photocatalytic reactions.

## 1. Introduction

Environmental protection and the mitigation of present problems caused by pollution are the fundamental criteria for sustainable development and, consequently, an improvement in life quality. In this context, it is important to highlight the care and protection of water, which is present directly or indirectly in all essential human activities, such as agriculture, industry, and energy generation. Industrial wastewater generally has high levels of pollutants, which, depending on their chemical composition, tend to be toxic, corrosive, and consequently harmful to the environment and the health of the population [1,2,3].

Wastewater from industry generally presents organic pollutants with different types of composition, resulting from different industrial activities, whether petrochemical, chemical, textile, pharmaceutical, agrochemical, cellulose, or food. Thereby, investing time in the development of new technologies that add more sustainable solutions than the current ones is urgent. In addition, it is necessary to develop methods for water purification that can help to mitigate this problem [1,2].

Some substances, called emerging pollutants (EP) have gained great attention in the context of studies from the last few years, due to the high toxicity and inefficiency of treatments for effluents contaminated with such substances. EP are substances that are part of several industrial sectors; however, in general, EP are not regulated with regard to the toxicity of their disposal and have great potential to impact human and environmental health [4].

EP can be evidenced by some groups of pollutants, among which are some natural and synthetic hormones, pesticides, plasticizing additives, industrial and food preservatives, cosmetics, flame retardants, fuel additives, and pharmaceuticals [5].

Despite extensive studies for wastewater decontamination, a large number of toxic substances and their combined use, especially those classified as emerging pollutants, induce the constant need to expand studies and seek more efficient forms of treatment aimed at mitigating the environmental impacts caused by these substances. Thereby, conventional water and effluent treatments cannot be efficient against most EP and, it is necessary to constantly search for increasingly effective and methodologies for the complete removal of these compounds from aquatic environments [6,7].

Rifampicin (RIF) is a drug with antibiotic action, used for the treatment of methicillin-resistant Staphylococcus Aureus infections [8]. RIF is one of the drugs adopted for the treatment of leprosy and tuberculosis, which is among the most widespread infections in the world [9,10]. The dosage required in the treatment of tuberculosis indicates the use of high doses of rifampicin for long periods, which is not fully absorbed by the patient’s body, is then excreted in feces and urine and consequently reaching urban effluents. In recent studies, significant concentrations of RIF were found in effluents treated in conventional treatment plants, indicating that the traditional mechanism of effluent treatment is not capable of degrading this substance [11].

Photocatalysis with semiconductors is considered a “green” process since it is one of the most promising methods for the efficient degradation of effluents with different types of contaminants [12]. In this way, it can be applied in an effective, sustainable way for the degradation of industrial wastewater of different possible applications, through a system that employs a semiconductor and irradiation of a light source [13]. To compose the photocatalytic system, many semiconductor oxides proved to be efficient to act in the degradation of contaminants in wastewater, such as TiO_2_, WO_3_, SnO_2_, and ZnO [14].

Zinc oxide (ZnO) has proved to be a good material to keep active in the next generation of photonic applications, due to its direct energy gap of 3.37 eV and a high electron binding energy of 60 meV [15]. Therefore, due to its high potential for photocatalytic activity, relatively low cost, and natural abundance, zinc oxide tends to be a semiconductor widely used in processes of contaminants photodegradation [16].

Nanoparticles of magnetic materials, such as manganese ferrite (MnFe_2_O_4_), are a growing object of study with wide applicability due to the easy manipulation of this type of material through magnetic fields [17]. The use of magnetic nanoparticles has great potential benefits, among which is the separation of materials through the application of a magnetic field, thereafter, the synthesis of materials composites in association with magnetic particles brings the possibility of a final separation of the material applied in a given process from its aqueous medium, minimizing residual damage from processes that act in such a way and also, by its own magnetic action, performs an immediate improvement in the catalytic properties of these composites in relation to the isolated material [18].

Hence, this work proposes the study of the photocatalytic degradation capacity of rifampicin by means of heterogeneous photocatalysis using ZnO/MnFe_2_O_4_ composites at different concentrations, in order to establish an analysis of the potential use of this treatment of effluents contaminated with PE, evaluating the effectiveness of the proposed procedure in decontaminating the effluent and promoting the residual removal of the applied material.

## 2. Materials and Methods

This study was divided into subsections of synthesis, characterization, and photodegradation. Table 1 below presents the reagents used in this study.

### 2.1. Syntheses

Zinc oxide (ZnO) was synthesized through a hybrid route, having a first co-precipitation step and a second calcination step. The co-precipitation step followed the methodology adopted by [19], using zinc chloride and sodium hydroxide acquired from Dinâmica Química, Indaiatuba—SP/Brazil, as precursors in the co-precipitation stage. The procedure was initially carried out with the preparation of solutions of the two precursors. Then, solution A (200 mL of 0.5 mol/L NaOH) was put under stirring and constant heating at 50 °C, after reaching the specified temperature, solution B (ZnCl_2_ 2.5 mol/L) was added by dripping. After the end of dripping, the solution obtained remained under stirring at 50 °C for another 90 min, where it remained at room temperature until it cooled down to be filtered and washed with deionized water. At the end of washing, the material was dried for 15 h in an oven at 60 °C. After the drying period in the oven, the material was calcined for 1 h at 400 °C.

Manganese ferrite (MnFe_2_O_4_) was synthesized through a hybrid route, having a first hydrothermal step and a second calcination step. The hydrothermal step followed the methodology adopted by previous studies [20], using manganese chloride and iron III sulfate as precursors. Next, 3.25 g of iron III sulfate and 1.0 g of manganese chloride in 50 mL of deionized water were added to a beaker. This solution was stirred at 200 rpm for 30 min. Then, the pH of the system was adjusted to 10 using an 8 M NaOH solution. With the pH adjusted and the precursor salts dissolved, the solution was transferred to a 100 mL hydrothermal autoclave reactor, which was closed and placed in an oven for 10 h at 150 °C. After 10 h, the reactor was placed on a bench for cooling at room temperature and then the dry material was taken to the calcination step for 3 h at a constant temperature of 500 °C.

The synthesis of the composite ZnO/MnFe_2_O_4_ took place through a hybrid route [21], where the process takes place in two steps, a co-precipitation step and a calcination step, having chloride as precursors zinc, sodium hydroxide, and manganese ferrite. In this process, the same steps of the synthesis of ZnO were followed, adding 1.0 g of manganese ferrite during the co-precipitation step, maintaining the same period of stirring and heating and going through washing and filtration before calcining for 1 h at 400 °C.

### 2.2. Characterizations

The synthesized materials were characterized using FTIR, SEM, and XRD. SEM analyzes were performed using a Scanning Electron Microscope (SEM), model VEGA3 TESCAN (Kohoutovice, Czech Republic). The samples, which are all in the form of powder, were placed in stub-type sample holders with a fixation on double-sided tape with a black background. The materials were analyzed with magnifications between 1000× and 10,000×, at a voltage of 10 kV.

Fourier transform infrared spectroscopy analyses were carried out in a Shimadzu FTIR IR PRESTIGE 21 spectrophotometer (Kyoto, Japan), in the spectral range from 4000 to 400 cm^−1^ with a resolution of 4 cm^−1^ operating in transmittance mode with 50 scans using an analysis method with KBr (potassium bromide). Where tablets with 1 mg of sample to be analyzed and 100 mg of KBr were prepared, in addition to a tablet with only 100 mg of KBr, corresponding to the “blank sample”.

X-ray diffraction analyses were performed using a Shimadzu DRX 6000 model diffractometer (Kyoto, Japan). The samples were added in the form of powder and analyzed in the range from 5 to 90° with an interval of 0.02° (2θ) using copper radiation (Cu) with an X-ray source, where this corresponds to a wavelength of 0.15406 Å, using a voltage of 40 kV and a current of 30 mA.

### 2.3. Photodegradation

The photocatalysis applications took place in a closed black box system with a mercury UV lamp (125 W), a magnetic stirrer, and a 500 mL borosilicate glass reactor (125 mm × 90 mm) uncovered was used for better dispersion and consequently more direct contact with radiation carrying a magnetic stirrer and a UV source. In this study, photocatalytic reactions were carried out with solutions prepared in 200 mL of deionized water with a constant concentration of Rifampicin of 10 ppm and varying the catalyst dosage by: 0.20, 0.40, and 0.60 g. Reactions were performed using the composite ZnO/MnFe_2_O_4_ and using pure ZnO. In all reactions, the time and sampling protocol was the same, with a total duration of 120 min, with the first 30 min without the incidence of UV light, called the dark period. Aliquots taken for analysis were collected at −30 (dark), 0, 10, 20, 30, 60, and 90 min. Then, these aliquots were taken to a centrifuge for 20 min at 20,000 rpm. After separation, the aliquots of the solutions were analyzed in a spectrophotometer UV-Vis Shimadzu, model UV-1800, with a 1 cm quartz cuvette, where decontamination was evaluated at the main wavelength of Rifampicin, 332 nm.

## 3. Results and Discussions

### 3.1. Characterizations

#### 3.1.1. Fourier Transform Infrared Spectroscopy (FTIR)

**Figure 1:**
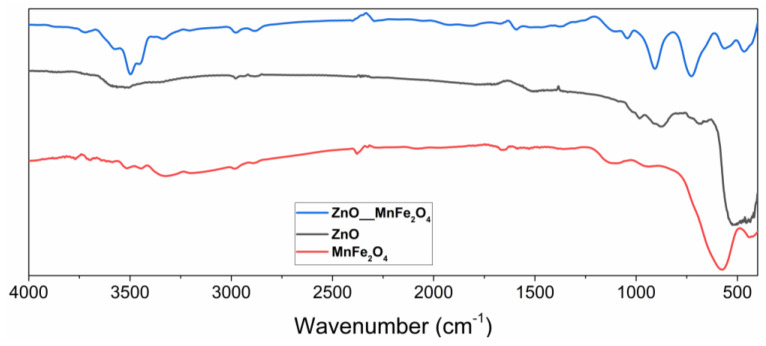
Spectrum in the infrared region of ZnO, MnFe_2_O_4_, and the composite ZnO/MnFe_2_O_4_ synthesized.

Figure 1 below shows the spectrum in the infrared region of the materials synthesized in this study.

Fourier transform infrared spectroscopy was performed in the region of 400–4000 cm^−1^ where, in the zinc oxide spectrum, the presence of characteristic Zn-O bands in tetrahedral coordination was evidenced [22]. The highest intensity band that appears between 400–600 cm^−1^ is characteristic of the stretching of the Zn-O bond in wurtzite structures, the most common crystalline form of the oxide.

The lower intensity band that appears at 670 cm^−1^ is also characteristic of the stretching of the Zn-O bonds [22]. The formation of bands is observed in the regions 750, 850, and 1000 cm^−1^, indicating stretching vibrations of the Zn-O bonds when in tetrahedral coordination [23,24,25]. In the region around the wavelength of 3500 cm^−1^, a band of low intensity is observed, which can be attributed to the stretching of O-H bonds, which have already been observed in studies involving materials of the same nature and which are generally related to water adsorbed by the surface in the analysis [26].

The readings of low-intensity vibrational bands observed in the region of 1100 cm^−1^ can be attributed to the presence of carbon dioxide, while the vibrational bands at 1650 cm^−1^ can be associated with the elongation of C-H bonds, which tend to occur in the infrared spectra of ZnO, due to the presence of residual impurities from the synthesis or from the manipulation of the synthesized material, due to the ease of surface adsorption of this material [26,27,28].

In the FTIR analysis of manganese ferrite, it was possible to observe bands manifested with characteristics that tend toward the presence of the synthesized material, such as the intense band in the region of 550–700 cm^−1^, characteristic region of reading ionic bonds of a metallic ion with oxygen, in which case it can be associated with Fe-O bonding, and Mn-O bonds generally seen in infrared spectra of ferrites [28,29].

The low-intensity bands observed in the regions of 1350–1480 cm^−1^ and 3200–3400 cm^−1^ can be associated with the adsorption of water, promoting the reading of stretching of O-H bonds, which tends to occur in this material, as observed by other works involving manganese ferrite [27,30].

The spectrum in the infrared region of the composite ZnO/MnFe_2_O_4_, clearly evidenced the characteristic bands of this material in the regions associated with the precursor materials. The band in the regions of 450–550 cm^−1^ and 570–650 cm^−1^ can be associated with the Zn-O, Fe-O, and Mn-O bonds, [23,24,31] respectively, being these regions where these connections tend to manifest, and they were observed in the materials synthesized separately in this study. The low-intensity bands presented in the regions of 1350–1650 cm^−1^, 2900 cm^−1^, 3050 cm^−1^, and 3480–3600 cm^−1^ can be attributed to vibrations of O-H bonds, coming from the surface adsorption of water in these materials, as observed in works that characterized materials with similar compositions [30,32].

In studies involving the synthesis and characterization of zinc oxide composites with other materials that have Fe-O bonds, spectra of great similarity were obtained, which indicates that the interaction of these materials tends to manifest bands related to the same types of bonds [31,32].

#### 3.1.2. Scanning Electron Microscopy (SEM)

**Figure 2:**
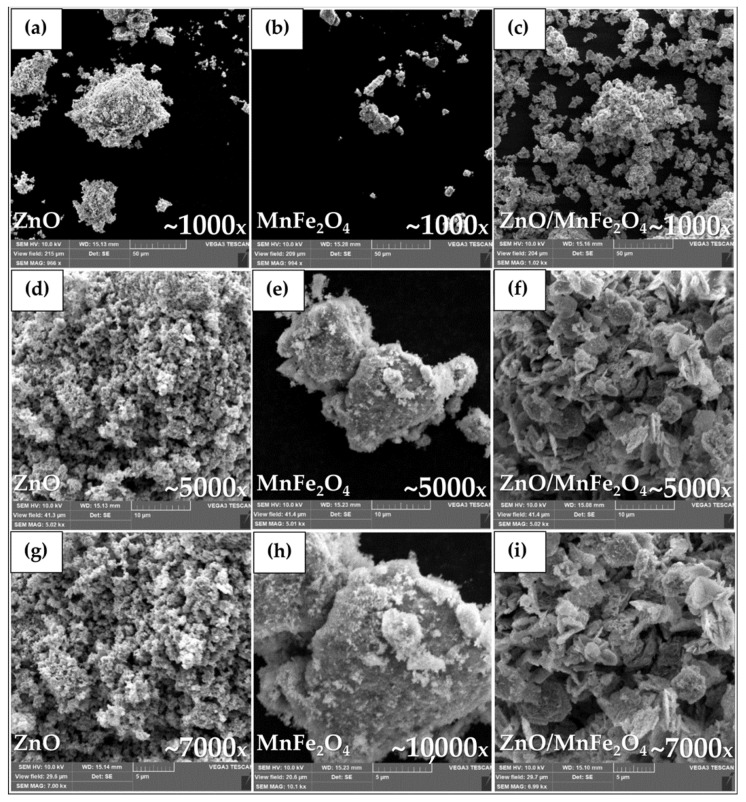
Micrographs (SEM) of (**a**) ZnO~1000×, (**b**) MnFe_2_O_4_~1000×, (**c**) ZnO/MnFe_2_O_4_~1000×, (**d**) ZnO~5000×, (**e**) MnFe_2_O_4_~5000×, (**f**) ZnO/MnFe_2_O_4_~5000×, (**g**) ZnO~7000×, (**h**) MnFe_2_O_4_~10,000×, (**i**) ZnO/MnFe_2_O_4_~7000×.

The ZnO micrographs in Figure 2 show in all highlighted magnitudes, a morphological formation characteristic of wurtzite, the predominant crystalline phase of zinc oxide, in which the formation of zinc oxide crystals occurs with large agglomeration, with irregular rounded geometry [33,34]. In Figure 2c, where the field magnification of approximately 7000 times is displayed, it is possible to observe some hexagonal crystals, which can be associated with the simonkolleite (SK) phase, a naturally occurring hydrated phase of zinc oxide, which made the intermediate part of ZnO synthesis.

During the synthesis performed, the Simonkolleite (SK) is transformed into ZnO through the heat treatment of calcination. The occurrence of characteristic crystals of this phase in the micrographs indicates that the calcination time was not sufficient to completely change the SK into ZnO [19]. This SK remnant in the material does not indicate a problem for the proposed application, since the generated radicals are of the same nature as ZnO and, as such, have photocatalytic potential [19,35].

In the micrographs presented in Figure 2, it is possible to observe the heterogeneity of the morphology of the MnFe_2_O_4_ particles, where crystalline formations and amorphous formations are visible, and where the agglomerates have sizes smaller than 5 µm.

This non-homogeneous agglomerated dispersion can be attributed to the high surface attraction energy of the material, which tends to induce morphological changes in the solid consolidation process during its synthesis [31]. Figure 2 below shows the materials synthesized.

The micrographs of the composite obtained show crystals of varying morphologies, some more rounded agglomerates, others more hexagonal, and all with the appearance of being covered with smaller particles [20,21].

At this point, it is important to note that the synthesis of the composite was carried out by the insertion of manganese ferrite in a step of the synthesis of zinc oxide, before calcination, that is, where there was still a greater proportion of SK, in this way, the MnFe_2_O_4_ particles precipitated next to the SK, having it on its surface and therefore in its forms.

The micrograph of Figure 2i shows the presence of a relevant fraction of hexagonal crystals, higher than that observed in the micrographs of pure ZnO, which indicates that the presence of MnFe_2_O_4_ delayed the formation of ZnO in the calcination process.

#### 3.1.3. X-ray Diffractometry (XRD)

**Figure 3:**
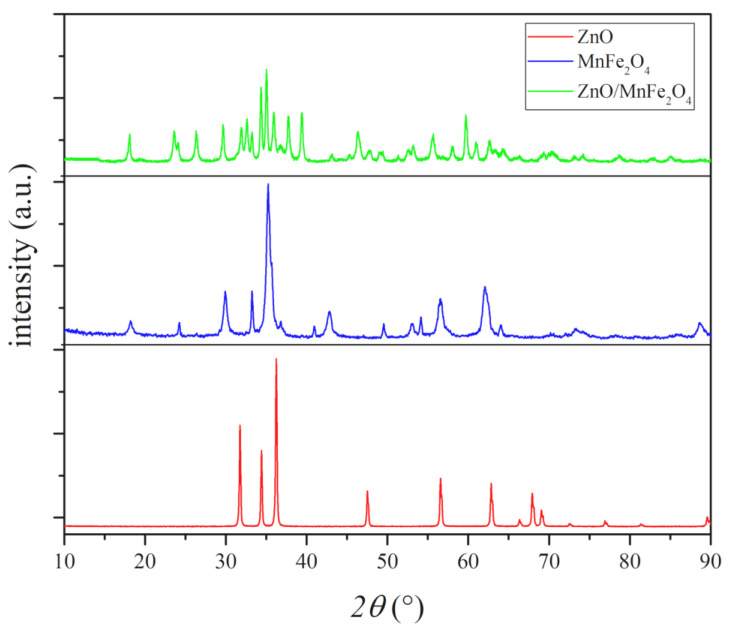
Diffractograms of the materials synthesized.

Figure 3 below presents the diffractograms of the materials synthesized in this study.

The synthesized ZnO diffractogram presents all peaks, with position, intensity, and orientation compatible with the wurtzite structure of zinc oxide, single phase, identified as 100% of zincite phase, with 13 highlighted peaks, as in ICSD 034477. The synthesized wurtzite zinc oxide structure presents parameters of hexagonal crystal lattice and organization, with results similar to previous works [34].

The experimental structure presented in the XRD of MnFe_2_O_4_ shows characteristic peaks of manganese ferrite with highlighted and well-defined intensity, and peaks with low intensity in regions characteristic of iron oxide phases, associated with hematite, an incidence commonly represented in ferrites, due to natural oxidation of the material [20,30,31]. After the identification of phases and the Rietveld refinement, it was possible to highlight the percentage of each crystalline phase identified, where a percentage of 93.69% of manganese ferrite and 6.31% of hematite was observed, which can be associated with oxidation by natural exposure of the material to atmospheric moisture [30,31]. The identification of the phases complied with the standards presented in the COD sheet 1528316 for manganese ferrite (MnFe_2_O_4_) and the ICSD sheet 082904 for hematite (Fe_2_O_3_).

The diffractogram of the composite ZnO/MnFe_2_O_4_ showed variations of crystalline phases formed between the constituent precursors, in addition to ZnO and MnFe_2_O_4_, it was possible to associate peaks with the zinc-manganese ferrite (ZnMnFeO_4_) phase, according to the JCPDF 89-7556 file and oxide, hematite phase (Fe_2_O_3_), according to ICSD sheet 082904. Through the Rietveld refinement, it was possible to measure the percentage of each crystalline phase present in the sample, where 27.64% of manganese ferrite was observed, 41.26% zinc-manganese ferrite, 21.65% zincite, and 9.45% hematite. Similar results were found in works involving the synthesis of materials with the same composition [20,31,36].

Another important piece of information regarding the experimental diffraction pattern of the synthesized materials is the average crystallite size, which can be obtained through the Scherrer Equation (1) [37].

The average size of the crystallite provides approximate data regarding the distribution of crystals in the sample, which is very important to evaluate the catalytic behavior of a semiconductor, since its contact surface influences its actuation mechanism and, consequently, the generation of oxidative radicals. The parameters involved in the methodology directly influence the size of the crystallite, such as the temperature and duration of the heat treatment [38,39].

Table 2 below presents the results of the average crystallite size for the three materials, calculated using the average of the five highest-intensity peaks. Similar results were found in studies that synthesized zinc oxide, ferrites, and composites among these materials, which intensifies the efficiency of the syntheses performed [36,38,39,40].

(1) $$L=\frac{k\;\cdot \;\lambda }{\beta \cdot cos\theta }$$

### 3.2. Photocatalytic Tests

**Figure 4:**
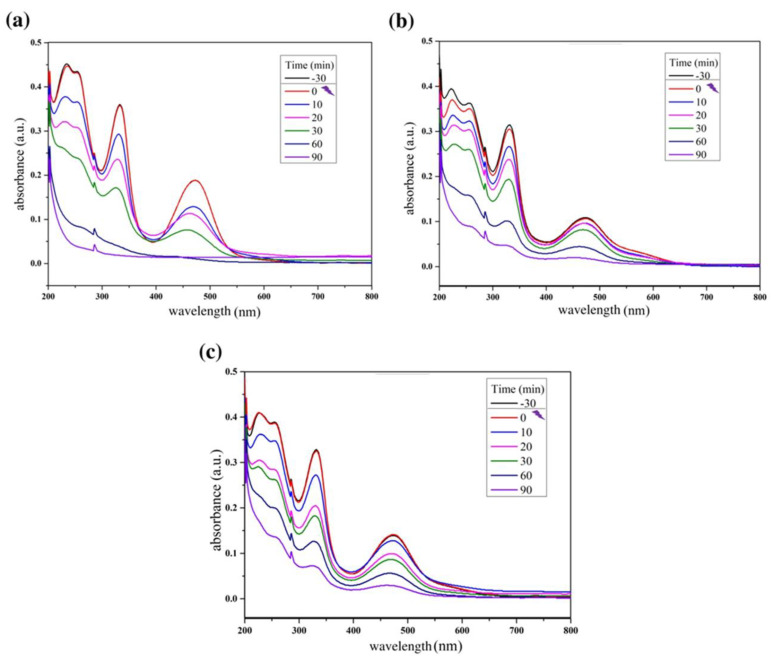
Reaction absorbance spectra of rifampicin solution during the photocatalytic tests using (**a**) ZnO: 0.20 g; (**b**) ZnO: 0.40 g; (**c**) ZnO: 0.60 g.

**Figure 5:**
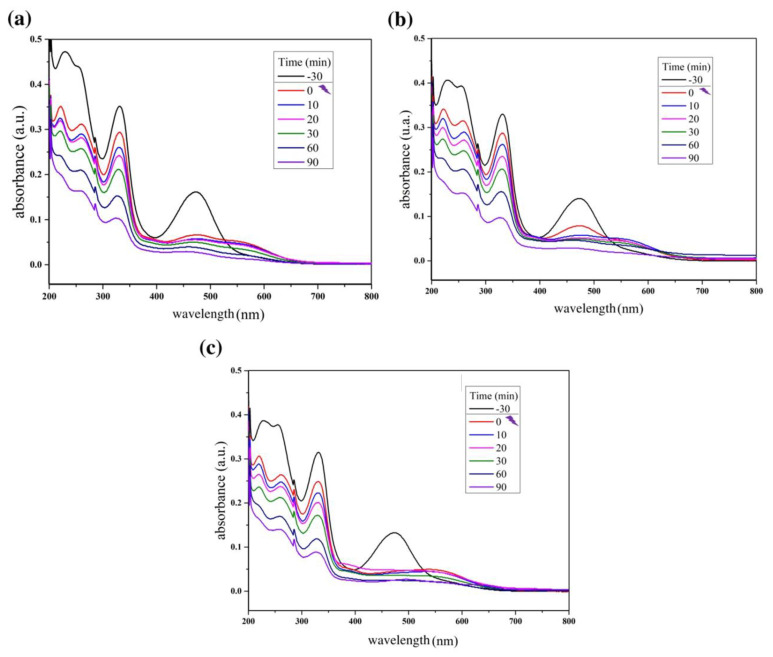
Reaction absorbance spectra of rifampicin solution during the photocatalytic tests using (**a**) ZnO/MnFe_2_O_4_: 0.20 g; (**b**) ZnO/MnFe_2_O_4_: 0.40 g; (**c**) ZnO/MnFe_2_O_4_: 0.60 g.

Figure 4 below shows the reaction absorbance spectra of photocatalytic tests using the ZnO synthesized in this study. Figure 5 shows the reaction absorbance spectra of photocatalytic tests using the composite ZnO/MnFe_2_O_4_ synthesized in this study.

**Figure 6:**
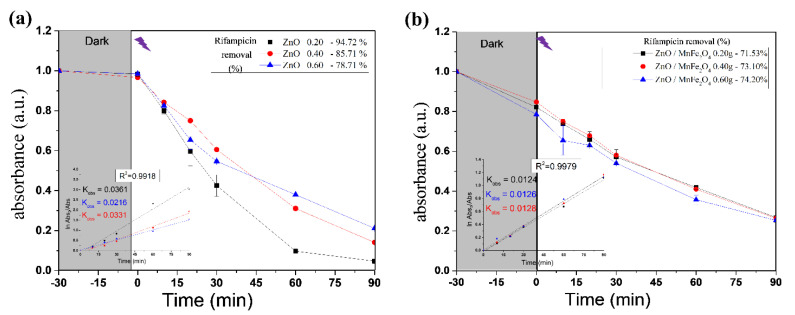
Rifampicin removal curves of photocatalytic tests by (**a**) ZnO; (**b**) ZnO/MnFe_2_O_4_. Inset—Pseudo-first order rate at time <90 min and *k_obs_* (min^−1^) as function of catalyst dosage.

**Figure 7:**
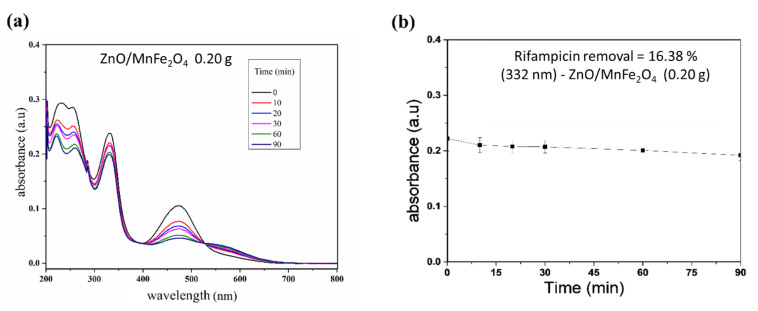
Control reaction in the dark. (**a**) Spectrum of rifampicin during reaction; (**b**) Removal curve of rifampicin.

Figure 6 shows the Rifampicin removal curves by the photocatalytic tests and Figure 7 shows a control reaction performed in the dark during the entirety of the reaction time.

The results shown in Figure 4 and Figure 6 provide information about the experiment performed with pure ZnO synthesized in this study. The first point to be observed is that during the dark period there was no reduction in the absorbance of the contaminant, Rifampicin, which indicates that the interaction of the oxidizing material with the contaminant solution did not produce adsorption. Another important point is to note that at the end of the cycle, the absorbance of the RIF was reduced considerably, reaching 94.72% in the best condition, which was 0.20 g of catalyst, analyzed by the most significant wavelength of the spectrum; at 332 nm, these notes indicate a great efficiency in the proposed photocatalytic system.

For the tests carried out with the composite ZnO/MnFe_2_O_4_presented in Figure 5 and Figure 6, a higher removal indicator is observed with the concentration of 0.60 g of catalyst, obtaining removal of 74.20%, analyzed at 332 nm, although this percentage is lower than the one obtained with pure ZnO, it is still of great significance, since the proposal of the composite is that there is the degradation and subsequent removal of residual solids through magnetic field induction [18,41]. In addition, Figure 7 shows that the maximum removal percentage without light was around 16% after 90 min. These findings corroborated the high photocatalytic performance either from the magnetic composite or its precursor, zinc oxide.

By estimating the values of the observed rate constant (*k_obs_*) by using Equation (2).

(2) $$\ln\left(\frac{{\left[A\right]}_{0}}{{\left[A\right]}_{t}}\right)=k_{obs}t$$

where [*A*]_0_ and [*A*]*_t_* represents the absorbance of RIF solution, measured at 332 nm at times zero and *t*, respectively; and *k_obs_* is the first-order oxidation rate constant (min^−1^). Fitting of the rate data using an order higher than one does not produce good correlation coefficients. Figure 6 presents the observed reaction rate constant values (*k_obs_*). When ZnO was used, the 0.2 g had the higher reaction rate, on the other hand, all catalyst dosages used from the composite generates similar removal rates [42].

### 3.3. Suggestion Mechanism of RIF Degradation by the Reusable Magnetic Composite

After the energetic activation of photocatalyst semiconductors by ultraviolet radiation, the high energy between the generated electron/gap mechanism and the obtained oxidative/reductive products (^●^OH, H^+^, O_2_^−^), generates progressive cycles of attack by breaking the radicals present in the molecular structure of Rifampicin (RIF), through steps (I), (II) and (III), to the parent compound and then repeatedly to the formed intermediates. Refs. [42,43,44], mainly by the action of the hydroxyl radical (^●^OH), which induces the breaking of bonds, when reacting mainly with oxygen, producing hydroperoxyls that will react with hydrogen ions and be removed from the structure in the form of H_2_O, generating carbon-centered radicals [43,45].

**Scheme 1:**
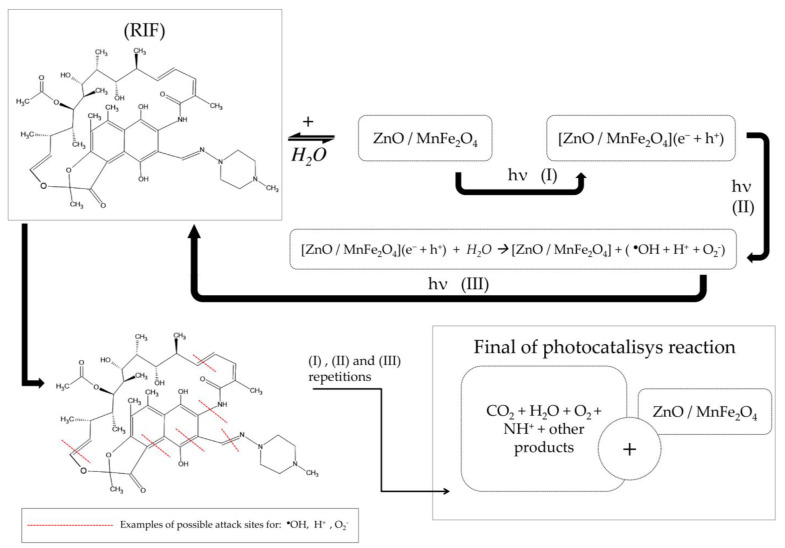
Suggestion of the mechanism of rifampicin degradation by the ZnO/MnFe_2_O_4_ composite.

The presence of a magnetic ferrite forming the proposed composite creates a secondary degradation mechanism, where Fe^2+^ ions react with hydrogen peroxide dissolved in the medium and produce Fe^3+^ ions and more hydroxyl radicals [42,46]. Fe^3+^ ions tend to be recombined in the process of energetic activation and creation and transition of electrons/holes, causing the degradation mechanisms involved to interact with each other and prolong and intensify the catalysis of compounds inserted into the aqueous medium [42,46,47]. A suggestion of the mechanism of rifampicin degradation by the ZnO/MnFe_2_O_4_ composite is shown in Scheme 1 below.

**Scheme 2:**
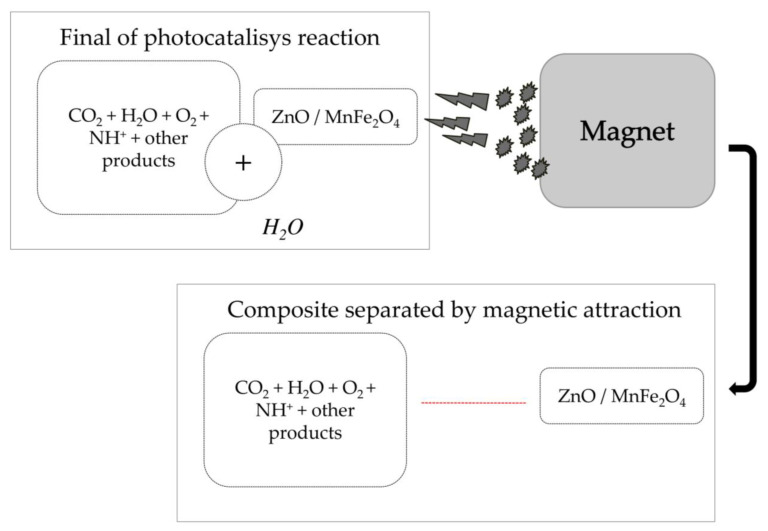
Application of magnetic field to remove catalyst composite dissolved in solution after reaction.

Another relevant point about the working mechanism of the composite used is the possibility of making the catalyst reusable by applying a magnetic field to remove the solid material dissolved in the water [41,48,49], as shown in Scheme 2 below.

## 4. Conclusions

The characterization of the materials in this study showed great efficiency in the syntheses carried out, where for ZnO a predominantly single-phase structure was obtained, for MnFe_2_O_4_ a structure with similar patterns to those of previous works was obtained and the composite presented characteristic links and phases of the interaction of the precursor materials in agreement with previous works that used these materials.

Regarding the photocatalytic tests, the results showed that the system employed has great efficiency in removing the emerging pollutant Rifampicin from aqueous media, where for pure ZnO a removal of 94.72% was obtained and for the composite ZnO/MnFe_2_O_4_ of 74.20%. It also verified the efficiency of removing residual solids from the reactions with composite through the application of a magnetic field, corroborating the validation of the efficiency of the proposed composite as a material for Rifampicin decontamination and corroborating the emphasis on promoting a composite catalyst, with secondary properties such as those discussed in this work, in which degradation is promoted by mutual mechanisms and the material proposed as a photocatalyst can be reused.

## Figures and Tables

**Table 1:** Summary of data on reagents used.

Name	Molecular Formula	Molar Weight (g/mol)	Producer	Purity Degree
Zinc Chloride	ZnCl_2_	136.60	Dinâmica	97.00%
Sodium hydroxide	NaOH	40.00	Dinâmica	98.00%
Manganese Chloride	MnCl_2_·4H_2_O	197.91	Êxodo Científica	97.00%
Iron III Sulfate	Fe_2_(SO_4_)_3_·xH_2_O	399.88	Neon	97.00%
Rifampicin	C_43_H_58_N_4_O_12_	822.94	AoPharmacêutico	97.00%

**Table 2:** Average crystallite size of the synthesized materials.

Average Crystallite Size *(La)*		
anode (Cu) λ		0.15406
Material	L	La
ZnO	50.97	51.75 nm
	50.98	
	53.31	
	50.98	
	52.54	
MnFe_2_O_4_	14.13	26.49 nm
	12.70	
	52.36	
	40.54	
	12.71	
ZnO/MnFe_2_O_4_	35.08	35.64 nm
	37.29	
	34.30	
	22.65	
	48.89	

## Data Availability

Not applicable.
